# Impact of using immersive virtual reality over time and steps in the Timed Up and Go test in elderly people

**DOI:** 10.1371/journal.pone.0229594

**Published:** 2020-03-13

**Authors:** Frédéric Muhla, Fabien Clanché, Karine Duclos, Philippe Meyer, Séverine Maïaux, Sophie Colnat-Coulbois, Gérome C. Gauchard

**Affiliations:** 1 UFR STAPS, Faculty of Sport Science, Université de Lorraine, Villers-lès- Nancy, France; 2 EA 3450 DevAH, Development, Adaptation and Handicap, Faculty of Medicine, Université de Lorraine, Vandœuvre-lès-Nancy, France; 3 OHS Centre Florentin, Nancy, France; 4 CHRU de Nancy–Neurochirurgie, Centre Hospitalier Universitaire de Nancy, Nancy, France; University of Rochester, UNITED STATES

## Abstract

Today, falls constitute a substantial health problem, especially in the elderly, and the diagnostic tests used by clinicians present often a low sensitivity and specificity. This is the case for the Timed Up and Go test which lacks contextualization with regard to everyday life limiting the relevance of its diagnosis. Virtual reality enables the creation of immersive, reproducible and secure environments, close to situations encountered in daily life, and as such could improve falling risk assessment. This study aims to evaluate the effect of immersive virtual reality by wearing a virtual reality headset with a non-disturbing virtual environment compared to real world on the Timed Up and Go test completion. Thirty-one elders (73.7 ± 9 years old) volunteered to participate in the study and the mean times and number of steps to complete a Timed Up and Go were compared in two conditions: actual-world clinical and virtual reality conditions. The results showed that the mean completion times and most of the mean number of steps of the Timed Up and Go in virtual reality condition were significantly different to those in clinical condition. These results suggest that there is a virtual reality effect and this effect is significantly correlated to the time taken to complete the Timed Up and Go. This information will be of interest to quantify the potential part of virtual reality effect on the motor control, measured in a virtual task using virtual controlled disturbances.

## Introduction

Falls are one of the greatest public health problems associated with aging and represent a significant human and financial cost for society. In France, about a quarter of people aged 65 to 85 declare falling every year [1], this rate being estimated at over 30% in industrialized countries [2]. Falls are also the direct cause of many complications, including fractures, functional decline, frailty, prolonged stays in hospital and institutionalization [3,4]. In addition, nearly 20% of people aged 55 to 85 declare a limitation of activity especially because of the fear of falling syndrome [5,6]. Falls are the result of a complex interaction of risk factors and as the number of risk factors increases the risk of falling and injury becomes higher [7].

The aging process can be considered as progressive alteration of motor, sensory and cognitive functions with advancing age. Frailty is usually accompanied by features such as sedentary lifestyle, decreased muscle strength, weight loss, osteoporosis, falls and fractures [7]. Age-related-physiological changes affect visual, vestibular and somesthetic functions, as well as posturo-kinetic reactions and central motor drives. Attentional resources, normally devolved to postural control, are shared during double task activities and the difficulty of a task depends mainly on the perceived difficulty of said task, implying a decrease in stability leading to falls [8], all the more important as this leads to fear of falling syndrome appearing [7]. In the elderly, the risk of falling will be particularly correlated to changes in the pattern of walking in comparison with healthy, younger people.

To evaluate a risk of falling, clinicians use tests and scales such as Timed Up and Go (TUG) [9], the Functional Gait Assessment [10], Tinetti test [11], One-leg balance test [12], Morse scale [13], or Berg Balance Scale [14]. These tests or scales offer balance or falling risk scores using situations that are generally simple and quick to implement, especially during a medical consultation. However, these assessment methods are considered by clinicians as first approach tests, aiming at identifying the elders which are most at risk of falling to direct them to specialized care [15]. In that way, the risk score based on a simple and fast application algorithm developed by CETAF (2009) [16] also serves this purpose. These clinical analysis methods have many advantages from a practical point of view to obtain data quickly and easily, but they are nonetheless decontextualized from daily life and integrate no multifactorial complexity of the fall, which could have an impact on the characteristics of screening tests in terms of sensitivity, specificity, positive and negative predictive values [17].

Monitoring the motor behavior of older people to assess their risk of falling should be done in a realistic environment with realistic tasks, whether indoor or outdoor. The problem is that such a test should be performed under the same conditions everywhere. However, the lack of precision on the TUG, for example, led to various interpretation and adaptations making results comparisons almost impossible. Thus, this study is interested to the virtual reality potential, because this type of new technologies, offer the possibility of a perfectly reproducible environment (visual and sound). To better contextualize a risk of fall assessment test, a method simulating what the individual goes through in daily living situations would consist of developing virtual reality (VR) *scenarii*. Among immersion displays available for customers, the development of virtual reality headsets allows a 360° 3D vision, navigation in and interactions with the virtual environment, providing a more immersive environment than previous gaming technologies [18–20]. However, VR requiring immersion and interaction with the virtual environment, it implies that the participant feels present in this immersive environment; thus, presence is a state of consciousness also defined as the subjective experience of being in one place or in an environment, even when it is physically located in another [21,22].

Among all clinical tests evaluating falling risk, the TUG is a commonly used screening tool in both the inpatient and community setting [23]. It stands out as being simple and fast to conduct as well as easy to interpret, it is recommended as a routine screening test for falls [24]. However, its low sensitivity (31%) and specificity (74%) could be limiting factors for using this test in predicting falling risk in the elderly [23]. Considering TUG and many other tests tend to ignore the daily life human-environment interactions and that immersion can provide contextualization, it could be conceived that VR immersion could impact motor control. In this respect, this feasibility study aimed to evaluate the impact of using immersive VR technologies on the motor control and feeling of presence in elder participants and especially to determine how virtual reality affects motor control by the time and the number of steps to complete a common task as the TUG with no additional virtual disturbances. One hypothesis is that, according to the enriched visual environment related to immersion, a discriminative effect of VR on the main parameters of TUG is expected, all the greater since individual feels present in the 3D immersive experience.

## Materials & methods

This study was conducted as part of a broader study of risk factors for falls in the elderly, which was approved by the "Comité de Protection des Personnes Grand Est III" (n°2018-A02637-48). Each patient has given their oral consent prior to the study. The data were analyzed anonymously.

### Participants

Thirty-one participants (20F-11M, age 73.7 ± 9 years old), autonomous and living at home, were recruited at the Rehabilitation Center Florentin (Office d’Hygiène Sociale of Meurthe-et-Moselle) in Nancy (France) at the end of their rehabilitation process. They volunteered to participate during one of their last days or visits in the center, just before being discharged from the rehabilitation center. None of them fell during the previous 12 months and their hospitalization followed, for the most part, the implantation of a knee, hip or shoulder prosthesis. As suggested by Podsiadlo [9], walking aids were accepted: nine subjects used a cane and seven other subjects a crutch. None of them had previous experience using an immersive virtual reality device.

### Tasks to perform

Participants performed three TUG trials (condition 1) and three TUG VR trials (condition 2), starting randomly from one condition or the other. The procedure of TUG was identical to that described by Podsiadlo [9], i.e. sitting comfortably on a chair (two armrests and seat about 46 cm height), back against the backrest, forearms on the armrests, walking aid in hand, feet on the ground for the initial position and, on the investigator’s signal given by the investigator, the participant must get up and walk at a comfortable and safe speed to a line created with adhesive tape glued to the floor 3m away from the chair, turn around and then return to sit down.

To provide spatio-temporal parameters (time and number of steps), a video recording (1920*1080p, 60fps, time displayed on the video player, cut to the closest frame) was made during the TUG test: its analysis allowed the TUG to be studied as a whole but also according to its 5 specific phases which are 1) Get up (GU): starts when the back gets off the backrest, 2) Go: starts when the participant is standing and starts walking, 3) Turn around (TA): starts when the participant starts to turn (when shoulders frontal plan starts to turn), 4) Return (Re): starts when the participant initiates the gait in the direction of the chair and 5) Sit down (SD): starts when the participant starts to turn to sit down. The motor necessities and constraints of these different phases could reflect different difficulty levels to accomplish them. Thus, it seems that the easiest phases to perform are the Go and Return phases, using more automated walking. The two phases with intermediate difficulty are the Get Up and the Turn Around phases, the first requiring a sufficient level of strength in the lower and upper limbs, but with the possibility to use armrests, the second requiring balance maintaining in a rotation on the spot. The most complex phase is the Sit Down phase, because it involves the combination of both a half-turn to go in the right direction and sitting down also requires enough muscular strength to control the descent to the seat. For both TUG and TUG VR conditions, mean times of completion of the whole test and of its different phases (Get up, Go, Turn around, Return, Sit down) were compared, as well as the mean number of steps.

### Virtual reality devices and immersive scenario

The TUG VR was performed using a virtual reality set including a HTC Vive Head Mounted Display (HMD) (Framerate: 90Hz, 2160×1200 combined pixels, 110° field of view, 470 grams) and a Nvidia Geforce GTX 1070 GPU equipped PC to run the VR software smoothly.

**Fig 1:**
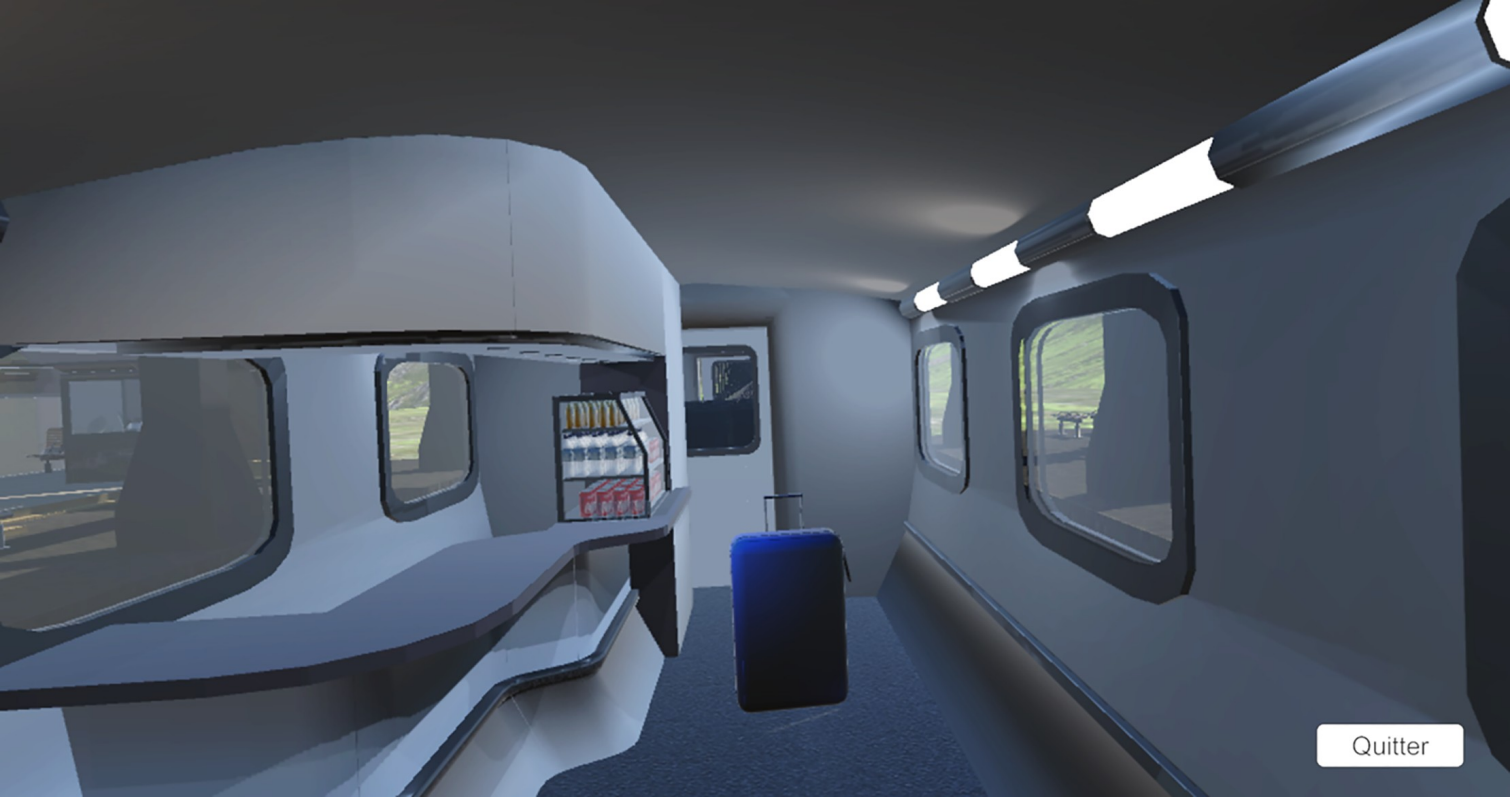
Virtual environment design.

The virtual reality TUG was designed on Unity game engine, simulating the interior of a stationary train bar car (Fig 1). The real chair was placed at the same spot as the virtual train seat and the line on the ground replaced by a blue suitcase to turn around in front of it. The reason for using a bulky piece of luggage is due to the limited viewing angle of the HMD, so participants can see the demarcation without having to bend their head too much [20,25].

Safety was guaranteed by the permanent presence of a physician close to the participant for every try, and by a collaborator dedicated to managing the headset wire, so it never interfered with the participant movements. Moreover, the virtual exploration was set in a larger real zone exempt of any obstacles, and the chair was kept in place by another collaborator.

### Presence evaluation for immersive experience

Presence was assessed using the IPQ (iGroup Presence Questionnaire) [26], translated from English to French by Isabelle Viaud-Delmon [27]. The purpose of this questionnaire is to subjectively evaluate the immersive nature of the experience offered by the "feeling of being there", according to a general question (G) and 13 other questions divided into 3 items which are spatial presence, involvement and realism. Spatial presence (five questions, SP1-5) refers to the feeling of being physically present in the virtual environment. Involvement (four questions, INV1-4) refers to the part of attention dedicated to the virtual environment rather than to the actual environment. Realism (four questions, REAL1-4) refers to the realism of the experience. With these items, the IPQ seems to be a good summary of the other questionnaires available [28]. The scores assigned to each question range from -3 to +3 and the scores for questions SP2, INV3 and REAL1 must be opposed for data processing [26]; a score near +3 reflects a high sense in the considered item.

### Statistics

The time and steps number distributions were tested with the Shapiro-Wilk’s W test and the distributions were not all considered as normal. They have been tested with the Wilcoxon test for dependent samples and presented as median and interquartile range. A probability level of p ≤ 0.05 was used as an indicator of statistically significant results. The results of the presence questionnaire are just presented using descriptive statistics (median and interquartile range of the scores from -3 to +3) and the correlation (Pearson, r) with the TUG VR time and TUG VR steps was tested too. Δ_*Time*_ is the difference of the time scores between the two conditions (*Time_TUG VR_−Time_TUG_*).

## Results

### Times

**Fig 2:**
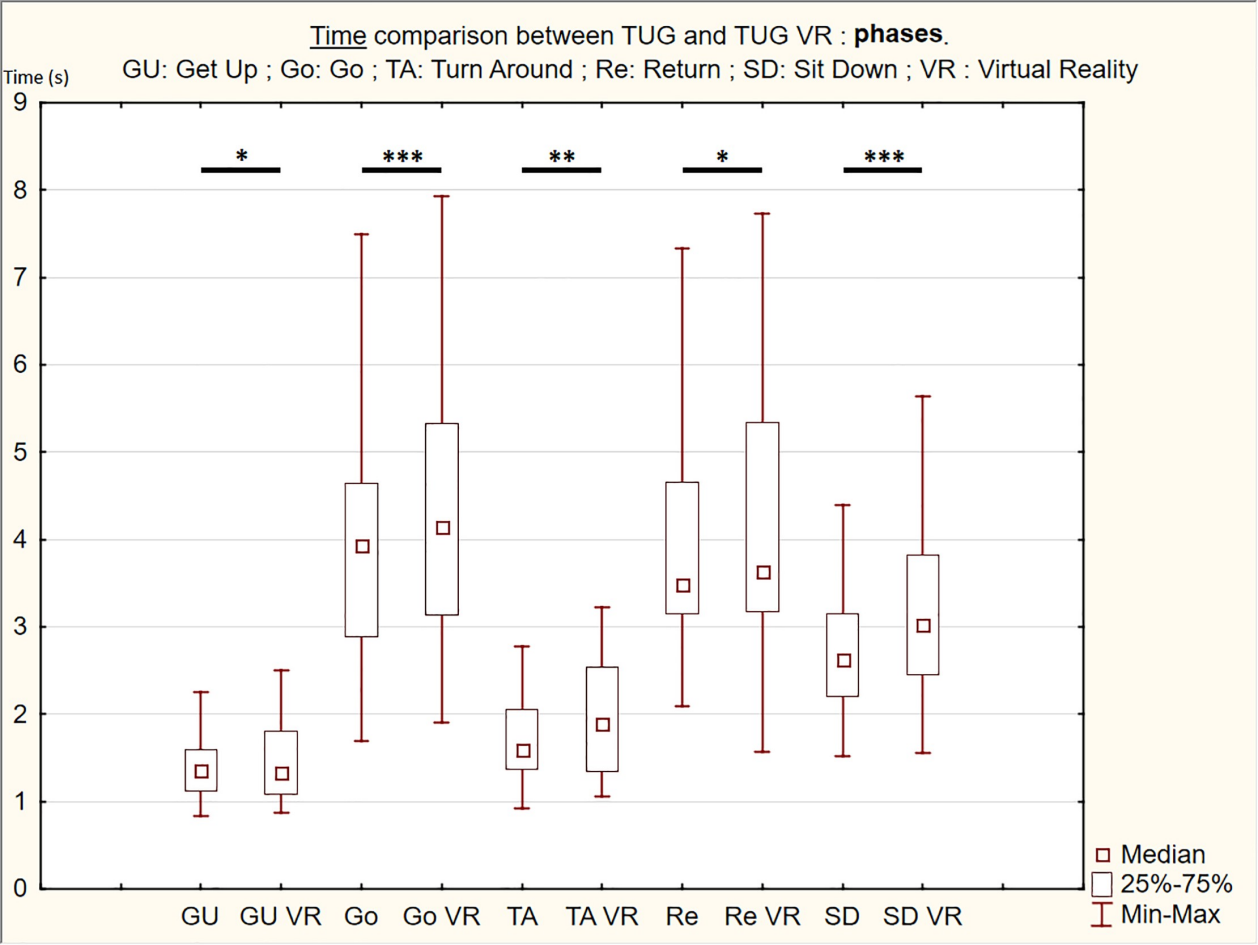
Box & Whiskers with Medians, Upper and Lower Quartiles, and Min-Max of the both conditions (TUG and TUG VR) mean times of the different phases. Wilcoxon test: *p<0.050; **p<0.010; ***p<0.001.

**Fig 3:**
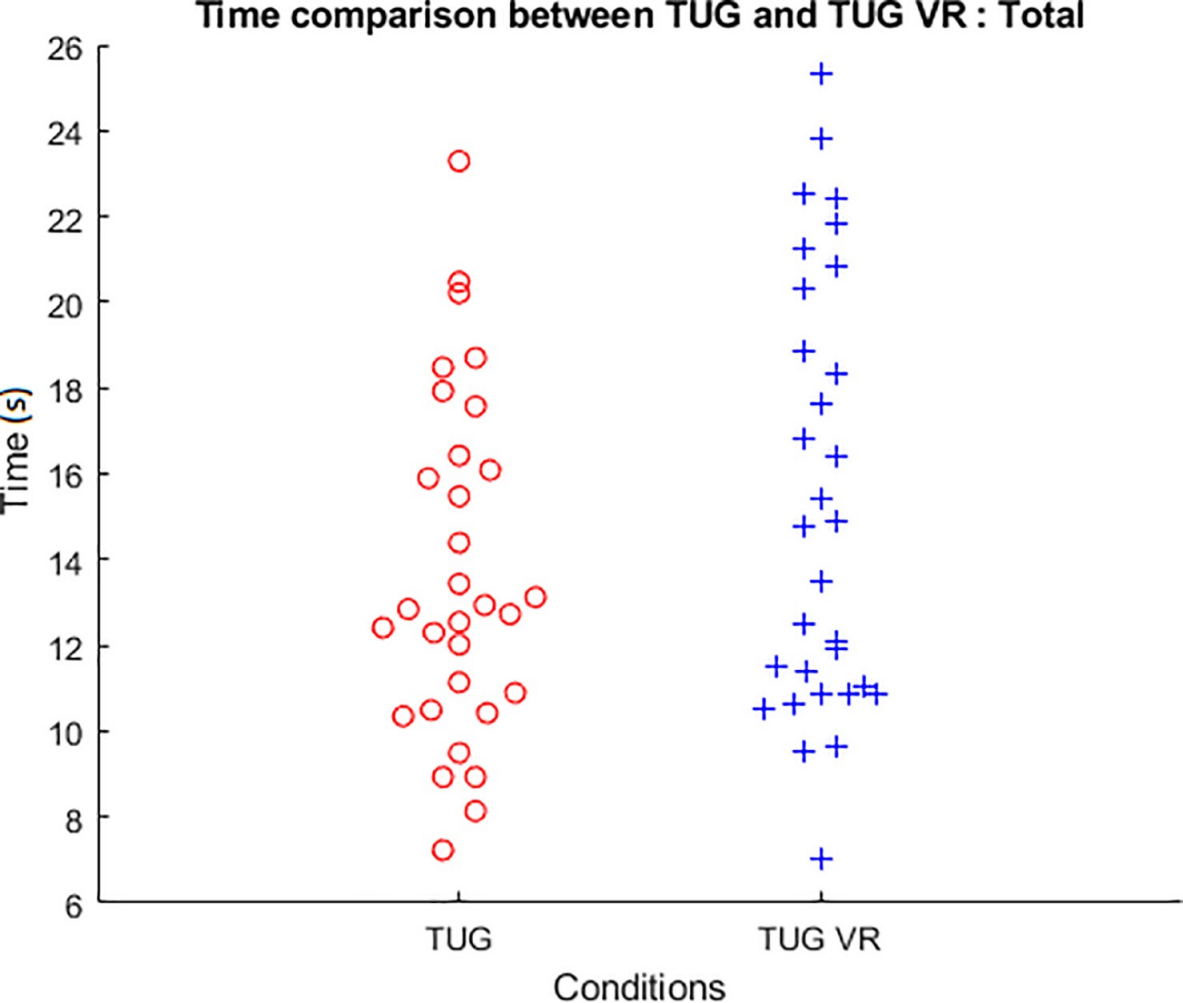
Bee swarm plot of total time in each condition (TUG and TUG VR).

The TUG VR condition showed an increase in the total completion time for the whole test and in completion times for each phase compared to the TUG condition, except for the Get Up condition for which time decreased (Table 1 & Figs 2 and 3). Statistically significant differences indicating higher time were observed for total time completion (z = 4.017, *p* < 0.001) and the main TUG phases (Go: z = 3.880, *p*<0.001; TA: z = 2.763, *p* = 0.006; Re: z = 2.567, *p* = 0.010; SD: z = 3.939, *p*<0.001). In contrast, statistically significant differences indicating lower time were observed for GU phase (z = 2.077, *p* = 0.038).

**Table 1:** Medians (interquartile ranges) of the times and number of steps in both TUG and TUG VR conditions, phases and total.

TUG Phase	Time (s)	Time VR (s)	Steps	Steps VR
Get Up (GU)	1.35 (0.48)	1.33 (0.72)		
Go	3.93 (1.75)	4.14 (2.19)	6 (2)	6.67 (2.67)
Turn Around (TA)	1.59 (0.69)	1.88 (1.20)	3 (1.08)	3.33 (1.42)
Return (Re)	3.49 (1.50)	3.63 (2.18)	6 (1.67)	6.33 (1.83)
Sit Down (SD)	2.63 (0.95)	3.02 (1.37)	3 (1.67)	3.33 (1.33)
Total	12.84 (5.56)	14.76 (8.63)	17.16 (4.83)	19.17 (6.5)

### Steps

**Fig 4:**
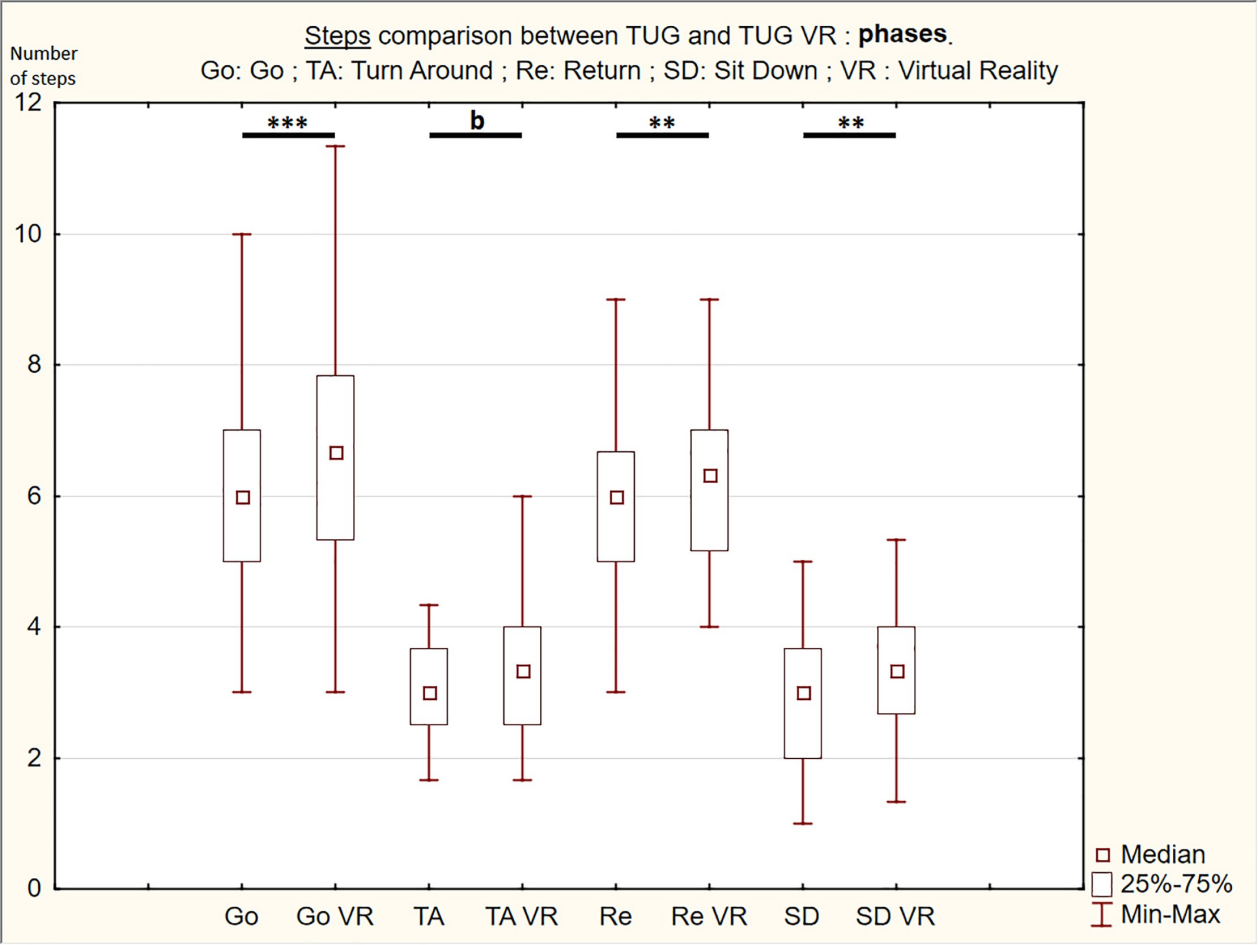
Box & Whiskers with Medians, Upper and Lower Quartiles, and Min-Max of the both conditions (TUG and TUG VR) mean number of steps for the different phases. Wilcoxon test: **p<0.010; ***p<0.001; ^b^p<0.100.

**Fig 5:**
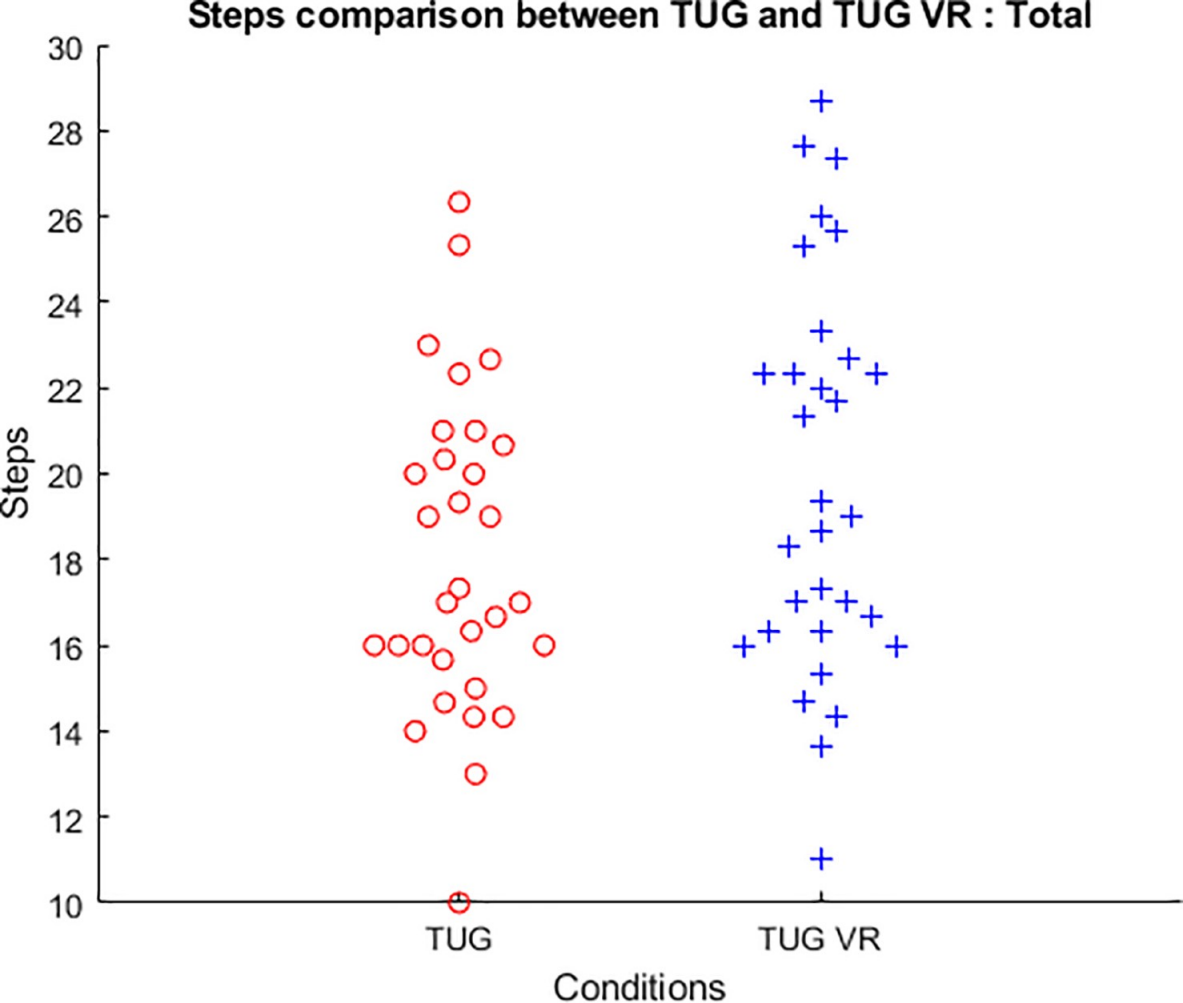
Bee swarm plot of total steps in each condition (TUG and TUG VR).

The TUG VR condition showed an increase in the total step number for the whole test and in the step number for each phase compared to the TUG condition (Table 1 & Figs 4 and 5). Statistically significant differences indicating more step were observed for total step number (z = 4.330, *p*<0.001) and the main TUG phases (Go: z = 3.829, *p*<0.001; Re: z = 3.271, *p* = 0.001; SD: z = 2.922, *p*<0.004). Borderline significance was observed for TA phase (z = 1.929, *p* = 0.068).

### Presence

**Fig 6:**
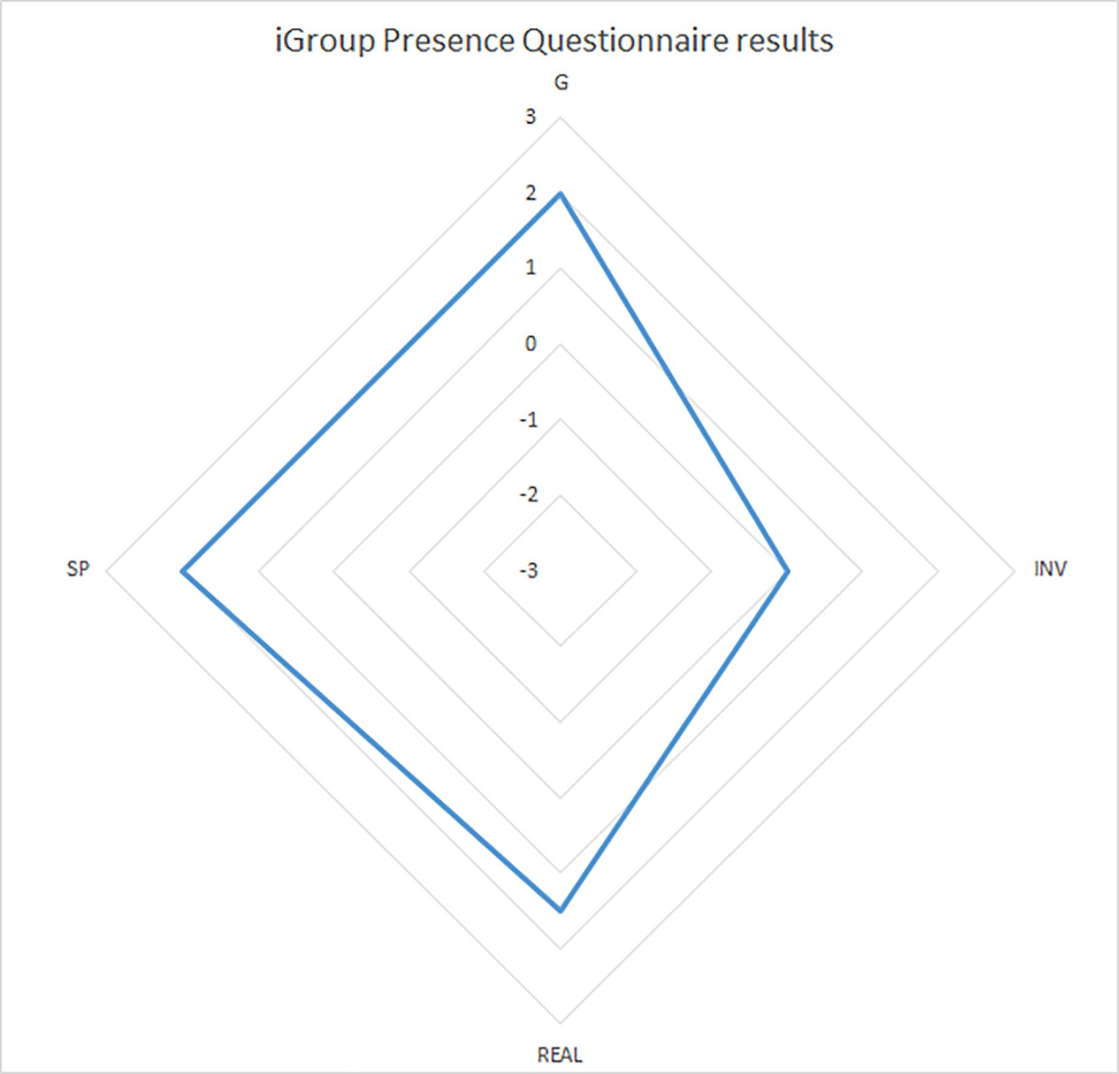
Graphical representation of the presence questionnaire results.

The results of the presence questionnaire showed, on a range from -3 to +3, median scores of (SP = 2 (1); INV = 0 (2.75); REAL = 1.5 (1.75); G = 2 (1.5)) (Fig 6). The general question and the 3 IPQ items have been compared to the total time and total number of steps of the TUG VR condition. There is no correlation between the time and the various items of the IPQ except for the general question (“In the computer generated world, I had the sense of being there.”), positively correlated (r = 0.36; p = 0.043): time increases as general question score increases and there is no correlation between the number of steps and the general question and the IPQ items.

### “VR effect”

**Fig 7:**
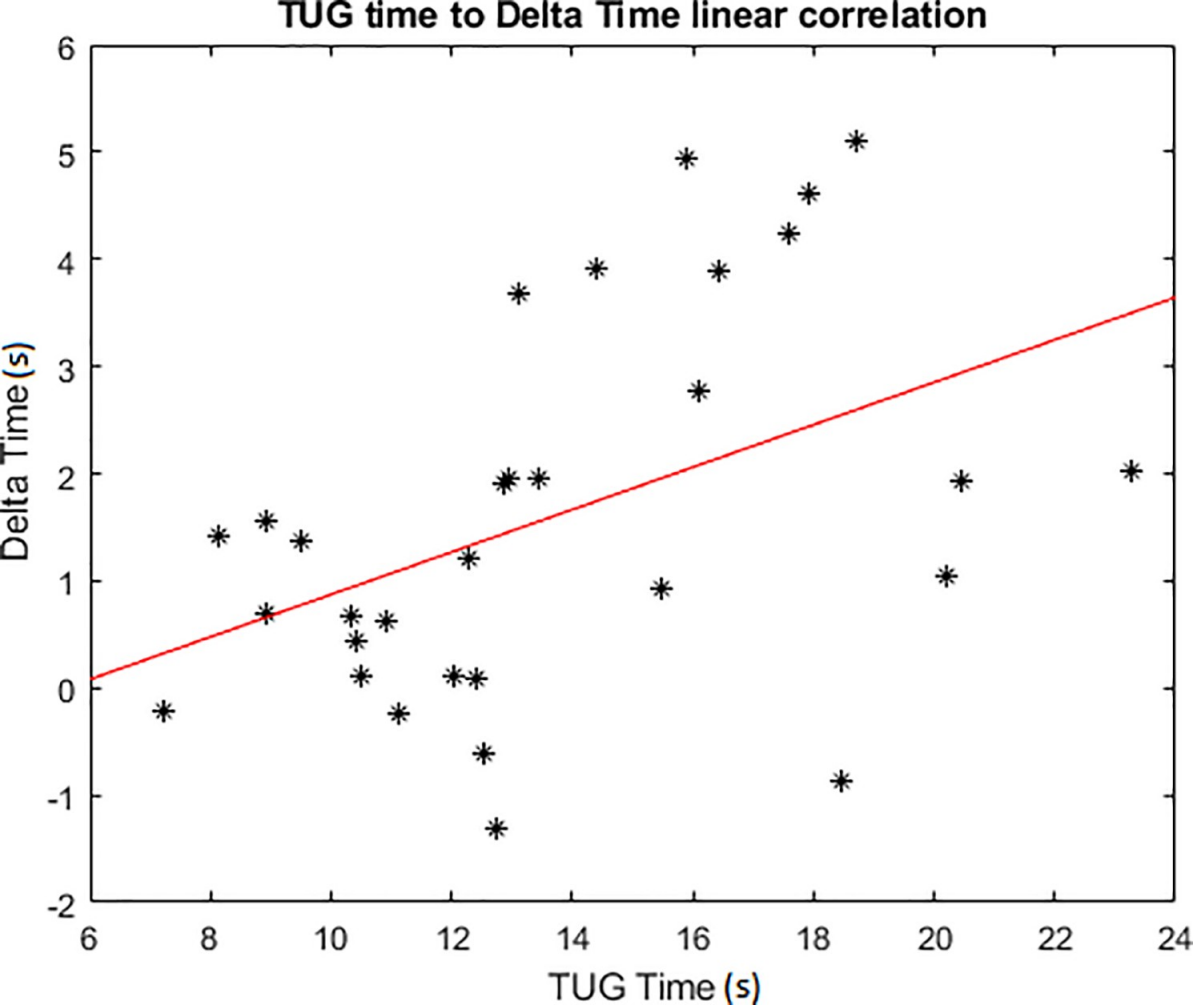
Scatter plot of the TUG total time correlation with Δ_Time_.

The results showed a difference between the VR and non-VR condition suggesting that there is a VR effect that needs to be quantified Δ_Time_ (Time_TUG VR_−Time_TUG_) is positively correlated to Time_TUG_ (p = 0.0122, r = 0.4445); if Time_TUG_ increases, Δ_Time_ increases too (Fig 7).

## Discussion

The objective of this study was to evaluate the effect of wearing a virtual reality head mounted display, immerging the user in a virtual environment free of added virtual disturbances. This effect was based on the time and steps required to perform the Timed Up and Go test and correlated to the level of “feeling of being there” in the virtual environment. This study showed significant differences between TUG and TUG VR for time and the number of steps in the entire test and for most of the phases. The time increased significantly in VR except for the Get Up phase (which decreased) and the steps number increased significantly in VR except for the Turn Around phase (not significant). The IPQ wasn’t significantly correlated to the time took to do the test except for the general question individually took (positively correlated).

The TUG test was designed as a binary test with a time threshold above which a patient has a high risk of fall. Ignoring the threshold, then the increase in test time becomes inversely proportional to the individual's ability to adapt to a situation that may compromise their balance. The thresholds usually found do not provide much sensitivity but the time score, as it increases, is meaning lower and lower functional mobility skills [9]. The hypothesis claiming that virtual reality simulating real-life could improve a falling risk test like the TUG is partially supported by the correlation between the general question of the IPQ and the time taken to complete the TUG VR by the participants. The results showed an increase in time and steps number as a response to a less secure environment than a medical office or a hospital corridor. Taking more time and using more steps can be seen as an adaptation strategy to ensure balance and avoid falling. However, even if the fear of falling was not evaluated here, participants may be afraid to fall without being able to see the real environment.

The presence questionnaire data are difficult to use because the results showed either an absence of or a weak correlation with the results. Therefore, it is necessary to break down the performed task as suggested in Methods. As a reminder, the TUG was considered as a succession of actions with varying levels of difficulty. The two phases of walking (Go and Return) would thus represent the simplest phases because they are relatively automated. The Get Up phase and the Turn Around phase would be considered as intermediate difficulty: The Get Up phase for the great solicitation of muscular strength and the Turn Around phase for the change of direction of 180° requiring specific skills to maintain balance. The most difficult phase to achieve is the Sit-Down phase because it combines two sub-actions: a half-turn and the sitting down itself, these two actions can, depending on the adaptation strategy used, be concurrent.

Ageing seems to affect gait parameters like speed, resulting in a decrease in step length and an increase in double-support phase time [29,30]. In healthy elders, gait with a decreased speed and a decreased step length can be interpreted as less destabilizing [31]. The variations observed in the walking cycles in time and in number of steps between TUG and TUG VR are intended to ensure stability. Ensuring an optimal level of security consists in transferring the objective of the dedicated task to achieve the task safely rather than achieving it quickly. The safety of the walking cycle then results in a gait using small steps, confirmed by the increase of the number of steps over the same distance, as well as a potentially increased duration of the double-support phase. Step width was not assessed, but it could have increased as well as the number of steps, in a safer balance maintaining strategy, especially in participants with impaired lower limbs [32]. The virtual environment has been designed in a train to provide a “realistic” experience allowing more TUG conditions with different visual and sound information. This environment can be obviously considered as a limit of the study because of its influence on gait parameters. In this respect, walking along the wall with windows on one side and a narrowing path on the other (Go phase) and still along the wall with a widen path (Return phase) could have impact the gait parameters. However, this type of conditions did not seem to impact locomotion except for participants with fear of falling [33]. In this study, we chose to use a versatile VR environment which can be enriched (running train, crossing a tunnel, wagon changing) and adapt itself to modify realization conditions of this sensorimotor task. Note that the two walking phases are located between more complex actions; the initiation of walking starts right after the chair lift or right after a half-turn and it ends, anticipating the half-turn or even, more complex, the Sit-Down phase [34]. This means that, in a 3 meters walking, first and last steps or cycles (out of approximatively 6 steps) are potentially influenced by the actions just before and just after [35], [36]. Moreover, it was observed that the number of steps taken during the walking phase was also dependent on the participants' anticipation for the half-turn (or half-turn + sitting) or rather the distance at which the participant starts their half-turn from the line or the chair. A strong anticipation of the half-turn involving a shortened walking path, the values of time and number of higher steps in VR thus reflect a lower anticipation on the half-turns. The difficulty of sitting on a real chair and virtually modeled could drastically reduce this anticipation. The anticipation of the half-turn phases in distance could perhaps become an interesting observable about adaptation strategies.

The Get Up phase time may have not change significantly because of the sensorial cues involved in the action and because the movement is ending with a bipedal stance where balance control, at least anteroposterior balance control, is not a hard challenge since participants have to start walking just after. In this phase, visual cues seem not as important as proprioceptive, vestibular and haptic cues. People used the armrest, and/or their walking aid in a symmetrical task to stand and start walking. The visual virtual environment provided by the VR headset had no significant negative effect on time. Even more, given the proposed set up in the virtual environment, worthless to say that the Timed Up and Go condition environment was not in a real train and it was done in a larger room. This implies that the closest visual cues were not in the same distance between the two conditions. Due to parallax, as the body sways, the closest visual cues provide more precise indications of amplitude and swing velocity than more distant clues and it has been shown, in posturographic analyses, that the closer the point of fixation of the gaze, the less the individual sways [37,38]. Thus, on the posture-locomotion adjustment plan, it was probably easier to sway less in the virtual environment than in the actual one.

The Turn Around phase changed in time but not in steps number in the VR condition, showing a high reproducibility of the strategy used to turn around. Notice that most participants used few steps to turn around, which is an observable often use to evaluate balance impairments. The use a few steps instead of a pivot for most participants, even in the TUG condition, showed difficulties in turning around smoothly due to global instability and probably fear of falling [39]. Other differences have been observed between TUG and TUG VR conditions in the Turn Around phase: some of the participants changed their turning side. Although we have not identified the dominant sides of the lower and upper limbs, it has been shown that laterality influences the turning side due to hemisphere dopamine imbalance. So, right-handers tend to turn to their left while non-right-handers (lefties and ambidextrous) tend to turn to the right [40]. However, it seems that a functional asymmetry can inhibit the effect of the dominant side for the direction of rotation (demonstrated in trans-tibial amputees) [41], biomechanical asymmetry then encountered in some of our participants. The few participants who changed their turning side chose to turn on their right in TUG condition and changed to turn left on the TUG VR condition. This could be explained by the virtual environment and the wall on the right. In this case, turning to the right means having the face close to the virtual wall and they probably preferred to keep sight on the other side to gather relevant information about their immediate environment. Note that walking aid could impact the way people turned. For example, when the crutch was located on the side of the half-turn, participants tended to turn around it. The effect of wearing a nearly half-kilo helmet in front of the eyes is questionable, especially for the case of a change of direction. Studies about additional “unbalanced” weight on the head, as well as an HMD, assessed the impact on neck angles and joint torques, finding various constraints in function of the head orientation. Literature showed that the search for visual cues during the U-turn may generate "extreme" rotations / flexions different from a condition without HMD. These "extreme" postures of the head over the trunk would be induced by both the limited field of view (FOV) and the mass of the HMD creating musculoskeletal stress. [25,42–44]. These effects seem to be limited in this study because of the low range of head motion during the U-turn. This point needs to be clarified in further studies which will try to thwart the cervical force momentum (balanced mass repartition or new strapping systems) and to assess participants FOV to consider the impact of the FOV reduction in the HMD. Combining head kinematics with eye-tracking could provide interesting information about head movements in order to compensate reduced field of view in visual exploration strategies. Testing various HMDs with different strapping systems in the same virtual environment could be interesting too to assess comfort and ergonomics improvements.

The last and most complex phase, the Sit-Down phase, was done in two different ways. The first, the fastest, was done by leaning and turning simultaneously and the second one, slower, was done as two independent phases: half-turn and sit-down. While looking at the participant behavior during this phase, there were people confident enough to sit right away and people who needed to touch the chair, searching the backrest/armrest by hand or touching the seat with the back of their knees before sitting on it. Referring to the TUG test, the more time you need to complete it, the more likely you are to fall. In this specific case, the comparison can be done between the two strategies used to sit-down where the fastest gives the best efficiency to the movement (optimal ratio between efficiency of the gesture and energy consumption) while the second one, the slowest, has a different objective which is to maintain its balance by simplifying the gesture to the detriment of the efficiency. Gait requires attention and the level of attention seems correlated to gait speed as it has been observed a decreased level of attention reducing gait speed [45], supporting the dissociation between half-turn and sitting down. In addition, anxiety about falling showed an increased demand of attention as postural threat increased resulting in lengthened double leg stance and shortened single leg stance but with a more conservative gait pattern [46].These results transposed to this Sit-down phase could explain that the level of anxiety generated by the VR condition where participants were not able to see neither their own body nor the real chair, increased the time of completion of this part of test as well as the other (go, turn around and return).

The presence questionnaire, despite its low correlations with the results, provides a state of the virtual experience at the time of the study. The results also depend on the user experience with immersive technology. Every participant in this study heard about virtual reality and VR headset but never tried it. After having collected the impressions of the patients, it seems that the VR experience has provoked in patients what can be described as “wow” or surprising effect. This observation shows that there may be a more or less pronounced gap between the existing and the already experienced by the subjects, especially for populations detached from new technologies.

The VR effect, determined by the time difference between the VR and the non-VR conditions, was positively correlated to the time taken to complete the non-VR test; so, the VR effect quantification has been based on information that the TUG provides. Beyond the time threshold determined with the optimal sensitivity and specificity, the time taken to complete the test could reveal the participant’s capacity to adapt and move into their environment. Because the instructions require the patient to walk at a secure pace, the longer the time, the harder to adapt to the environment and constraints is. This correlation suggests that participant easily interacting with their actual environment will be more comfortable in the virtual one.

However, we did not assess the impact of the gender effect on the locomotor control while using immersive VR. This could be the point of further studies since ageing effects may be gender related, we could observe different motor strategies related on various levels of osteoporosis or of fear of falling for example. In this study, we chose virtual reality to simulate reality, not an imaginary world. As we wanted to give a consistent experience to our participants, we chose a train environment which will be enriched in further studies and still be consistent. Thus, we will be able to test people in a virtually moving train as a pretext to add linear flow to the task. In the whole virtual experience, hardware and virtual environment were not disconnected and at this point we are not able to ponder their respective impacts on motor control. So matching real and virtual environments should be put to the test.

## Conclusion

This study comparing VR and non-VR conditions for the completion of a TUG, without adding any disturbance (real or virtual), has shown differences in time and steps number, meaning there is a “VR effect”. This VR effect, probably dependent on the current technology, must be quantified for further VR experimentation as it would be impossible to decorrelate VR effect from disturbance effect on a participant action. The VR effect, determined by the time difference between the two conditions, is significantly correlated to the TUG time. So, VR effect can be quantified with the time taken to complete the task in the real environment and it suggests that more easily the participants can interact with the real environment, the more comfortable they will be in the virtual one. More studies are required to use the TUG in a more clinical objective, correlating TUG VR parameters with prospective longitudinal fall follow-up, partly reflecting dependence and frailty degree in the elderly. Some issues had probably hurt the virtual experience such as the absence of vision one’s body and the actual environment or the sounds/noises of the actual environment decreasing the level of involvement. Further studies on motor behavior using VR should implement full body virtual representation and provide an even more immersive experience (visual, audio…) and test motor behavior with more complex interactions.

## Supporting information

S1 Data Time, number of steps and IPQ data. (XLSX)
